# Supplementary material for: Human RAD51 paralogue SWSAP1 fosters RAD51 filament by regulating the anti-recombinase FIGNL1 AAA+ ATPase
Source: Nat Commun. 2019 Mar 29;10:1407. doi: 10.1038/s41467-019-09190-1 (PMC6440994; doi:10.1038/s41467-019-09190-1)
Supplement: Supplementary file 1 — Supplementary Information [file 41467_2019_9190_MOESM1_ESM.pdf]

Supplementary Information

Human RAD51 paralogue SWSAP1 fosters RAD51 filament by regulating the anti-recombinase FIGNL1 AAA+ ATPase

Matsuzaki et al.

Supplementary Figure 1

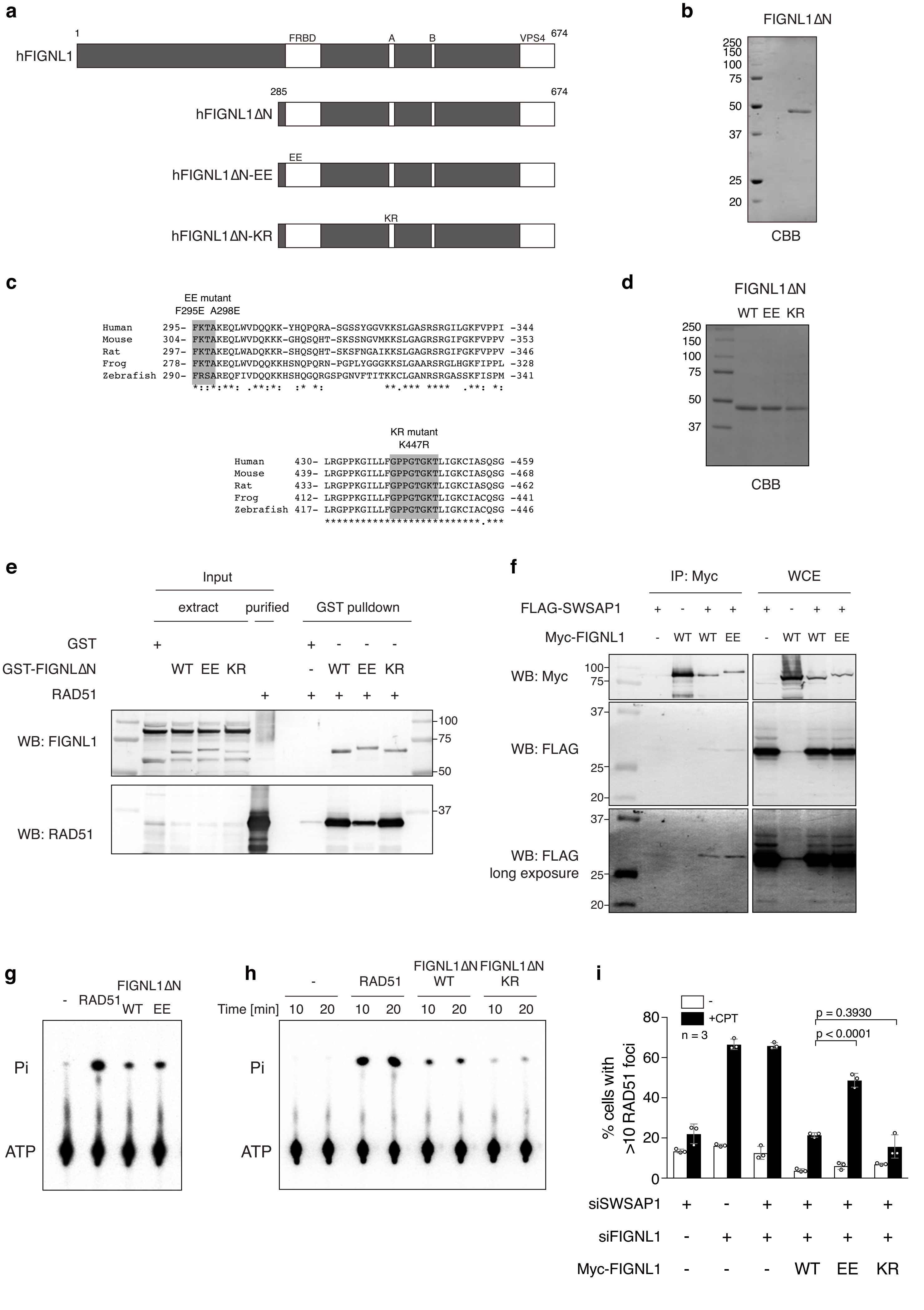

Supplementary Fig. 1 Functional analysis of FIGNL1 mutants

a Schematic representation of human FIGNL1, FIGNL1ΔN, FIGNL1ΔN-EE and FIGNL1ΔN-KR used in Fig. 4. **b** Purified FIGNL1ΔN was analysed by SDS-PAGE with Coomassie staining. **c** Amino acid sequence comparison of FIGNL1's RAD51 binding domain (FRBD)(Top) and Walker A motif (shaded region)(bottom). **d** Purified FIGNL1ΔN-EE and -KR were analysed by SDS-PAGE with Coomassie staining. **e** Interaction between purified FIGNL1 mutant proteins and RAD51. Bacterial extract expressing GST, GST-FIGNL1ΔN, GST-FIGNL1ΔN-EE or GST-FIGNL1ΔN-KR were incubated with glutathione beads. After wash, the beads were incubated with purified RAD51. Samples were eluted with glutathione and subjected to western blotting. **f** Co-immunoprecipitation analysis of FIGNL1-EE mutant. Myc-FIGNL1 or Myc-FIGNL1-EE were co-expressed with FLAG-SWSAP1 in 293T cells, and subjected to IP and analysed by western blotting with indicated antibodies. **g-h** ATPase activity of FIGNL1ΔN, FIGNL1ΔN-EE and FIGNL1ΔN-KR was analysed. [γ-^{32}P]ATP was incubated with indicated proteins. The products were analysed by thin-layer chromatography using PEI plates. The plates were analysed by the phosphor imager and its images are shown. **i,** After 96-h transfection of siRNA for FIGNL1 and SWSAP1 with the expression of siRNA-resistant FINGL1, FIGNL1-EE or FIGNL1-KR mutnat proteins, cells were treated with 100 nM of CPT for 22 h and immuno-stained for RAD51. Quantification of RAD51-positive cells was analysed. Data are mean ± s.d, (n=3, three biological independent). Statistical significance was measured by Mann-Whitney's *U*-test. Statistics and reproducibility; see accompanying Source Data.

Supplementary Figure 2

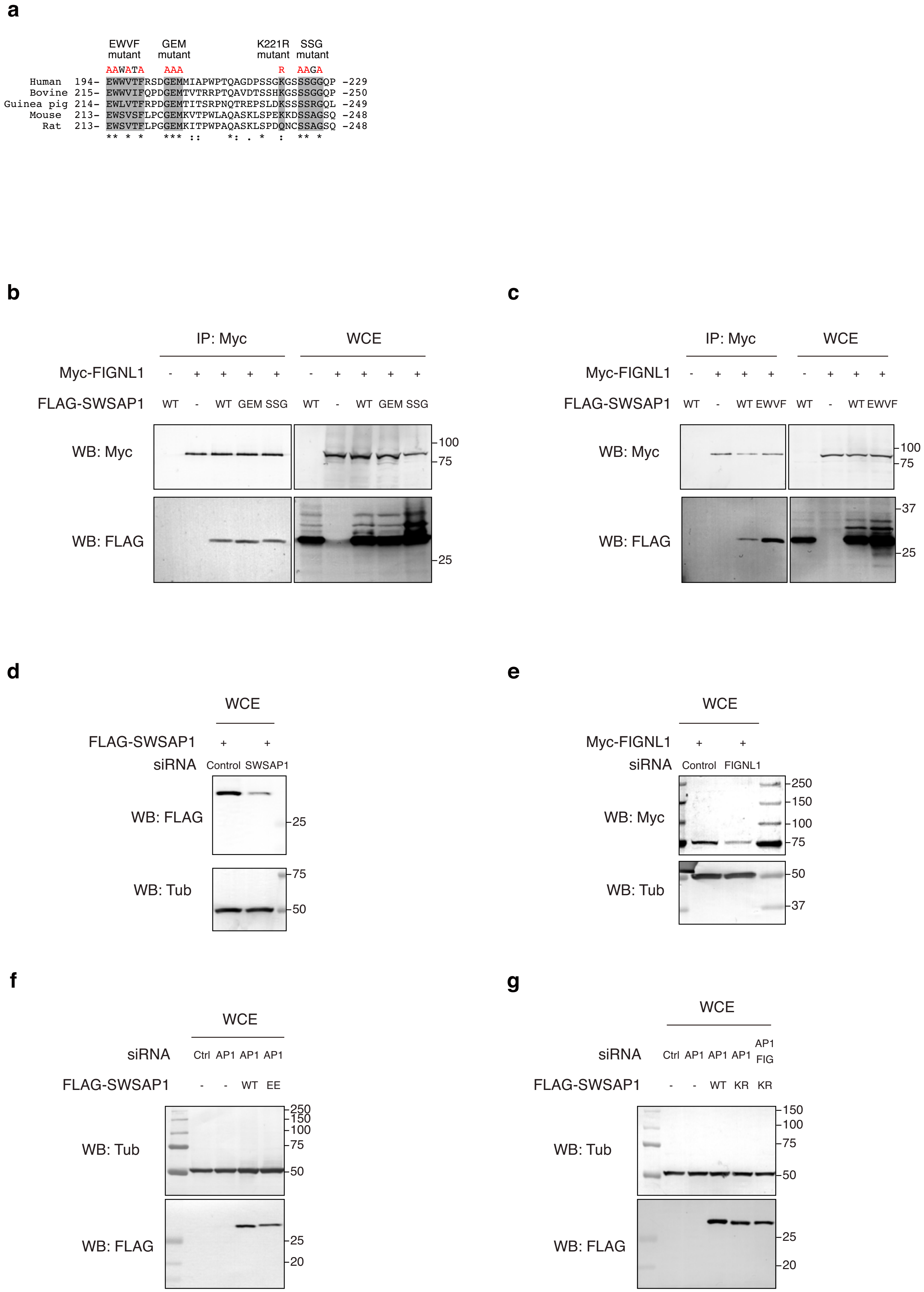

Supplementary Fig. 2 Interaction between SWSAP1 C-terminal point mutants and FIGNL1

a Amino acid sequence comparison of conserved SWSAP1 C-terminus. Amino acid substitutions (red) are indicated above the sequences. **b-c** Co-immunoprecipitation analysis of SWSAP1 mutants. Myc-FIGNL1 and indicated FLAG-SWSAP1 mutant proteins expressed in 293T cells were used for IP and analysed by western blotting with FLAG and Myc antibodies. **d** SWSAP1 depletion in FLAG-SWSAP1-expressing cells. Evaluation of the effect on siRNA for SWSAP1 in U2OS cells was analysed for FLAG-SWSAP1. Tubulin was an internal control. **e** FIGNL1 depletion in Myc-FIGNL1-expressing cells. Evaluation of the effect on FIGNL1 siRNA in U2OS cells was analysed for Myc-FIGNL1. Tubulin was an internal control. **f** Protein expression of siRNA-resistant SWSAP1 and SWSAP1-EE. Evaluation of the effect on SWSAP1 siRNA on siRNA-resistant for SWSAP1 and SWSAP1-EE in U2OS cells was examined. Tubulin was an internal control. **g** Protein expression of siRNA-resistant SWSAP1 and SWSAP1-KR. Evaluation of the effect on SWSAP1 siRNA on siRNA-resistant for SWSAP1 and SWSAP1-KR in U2OS cells was studied.

Supplementary Figure 3

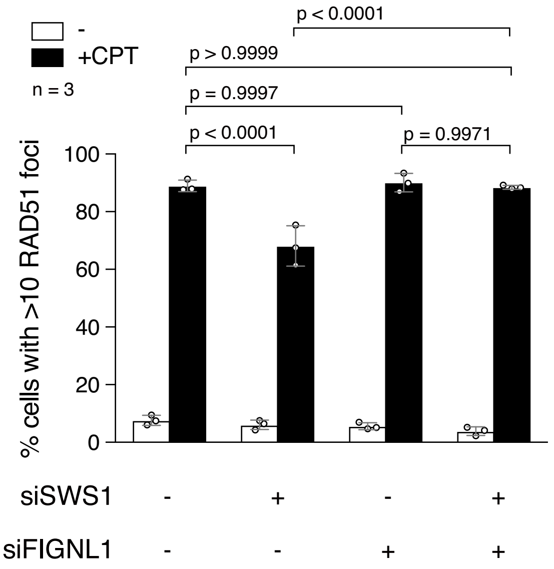

Supplementary Fig. 3 RAD51 focus formation defect in SWS1-depleted cells is suppressed by the FIGNL1 depletion

Immuno-staining analysis of RAD51 focus in control U2OS cells and SWS1-depleted cells with or without the FINGL1 depletion at 22 h after the treatment of 100 nM of camptothecin (CPT). Quantification of RAD51 focus-positive cells (more than 10 foci per a cell) in the indicated cell lines. At each point, more than 200 cells were counted. Quantification of RAD51 focus-positive cells in the indicated siRNA-transfected cells. Data are mean ± s.d. (n=3, three biological independent). Statistical significance was measured by Mann-Whitney's *U*-test. Statistics and reproducibility; see accompanying Source Data.

Supplementary Figure 4

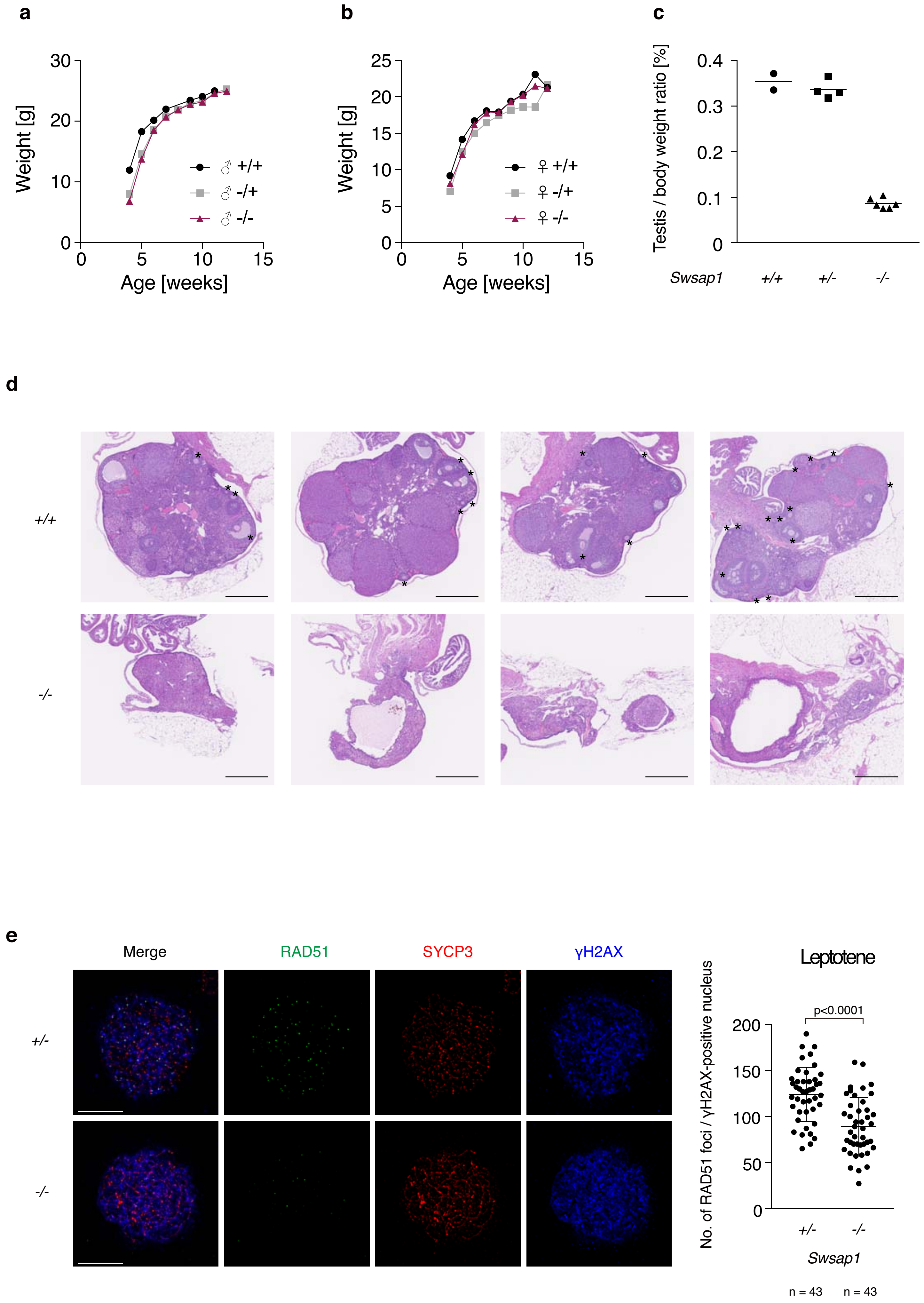

Supplementary Fig. 4 Meiotic defects and RAD51 assembly defects in *Swsap1*$^{-/-}$ mice

a Weight analysis of *Swsap1* male mice. Average weights of more than 3 mice for each group are shown. Error bars are not shown to render the graph readable. **b** Weight analysis of *Swsap1* female mice. Average weights of more than 3 mice for each group are shown. Error bars are not shown to render the graph readable. **c** Testis weight of wild-type, *Swsap1*$^{+/-}$ and *Swsap1*$^{-/-}$ mice. Testis weight was normalized by body weight. Mean is shown as a bar. **d** Cross sections of fixed ovary were stained with HE. Several representative images are shown. Asterisks denote developing ovaries. Bar 500 μm.**e** RAD51, SYCP3 and γH2AX immunofluorescence analysis of leptotene spermatocytes. Left, Representative images of *Swsap1*$^{+/-}$ and *Swsap1*$^{-/-}$ spermatocyte spreads are shown. Right, Quantification of RAD51 foci in γH2AX -positive leptotene spermatocytes. Bar 10 μm. Data are mean ± s.d. (n=43, three independent experiments). Statistical significance was measured by Mann-Whitney's *U*-test. Statistics and reproducibility; see accompanying Source data. Mean is shown as a bar.

Supplementary Figure 5

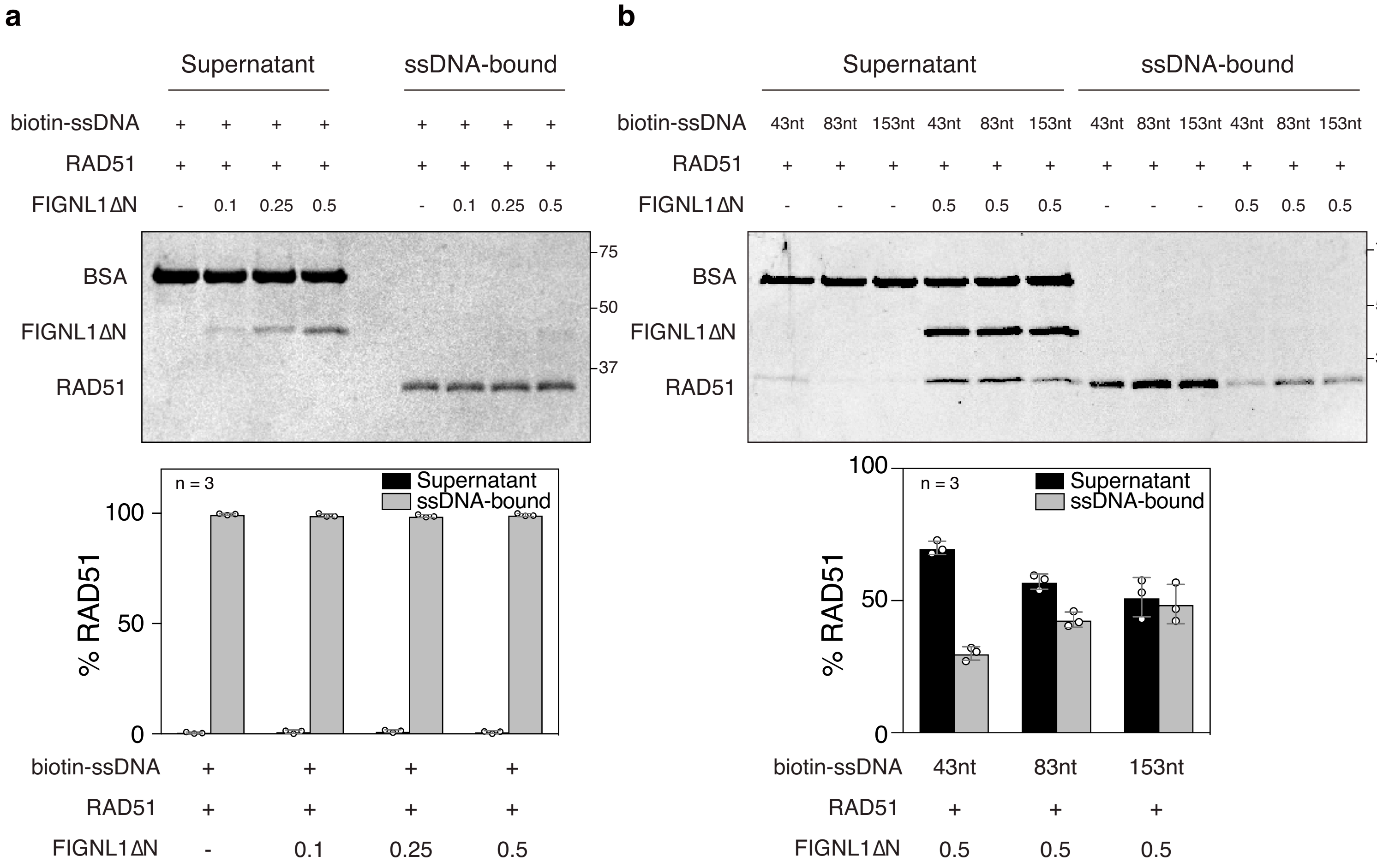

Supplementary Fig. 5 Property of FIGNL1's RAD51 filament disruption activity

a RAD51 disassembly assay in the presence of Ca^{2+}. ssDNA pre-bound to RAD51 in the presence of ATP and Ca^{2+} was incubated with an increased concentrations of purified FIGNL1ΔN. After 20 min, supernatants and bound fractions were recovered. Top, a representative SDS-PAGE gel for supernatants and bound fractions stained with CBB. Bottom, Quantification of dissociated RAD51 (Supernatant) and ssDNA-bound RAD51. Intensity of each band of RAD51 was quantified by Imager. The values of RAD51 bands in the supernatant or ssDNA-bound fractions were divided by the total value of RAD51 bands (both in supernatant and bound fractions). Data are mean ± s.d. (n=3, three biological independent). Statistical significance was measured by two-tailed *t*-test *U*-test. Statistics and reproducibility; see accompanying Source Data. **b** Effect of ssDNA length on RAD51 disassembly. RAD51 filaments formed on 43nt-, 83nt- or 153nt-ssDNA were incubated with 0.5μM FIGNL1ΔN. Top, a representative image of SDS-PAGE gel stained with CBB. Bottom, Quantification of dissociated RAD51 (Supernatant) and ssDNA-bound RAD51. Data are mean ± s.d. (n=3, three biological independent). Statistical significance was measured by two-tailed *t*-test *U*-test. Statistics and reproducibility; see accompanying Source Data.

Supplementary Figure 6a

Related to Fig.1a

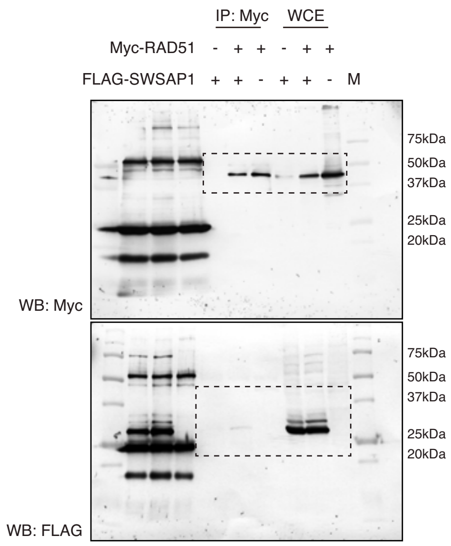

Related to Fig.1d

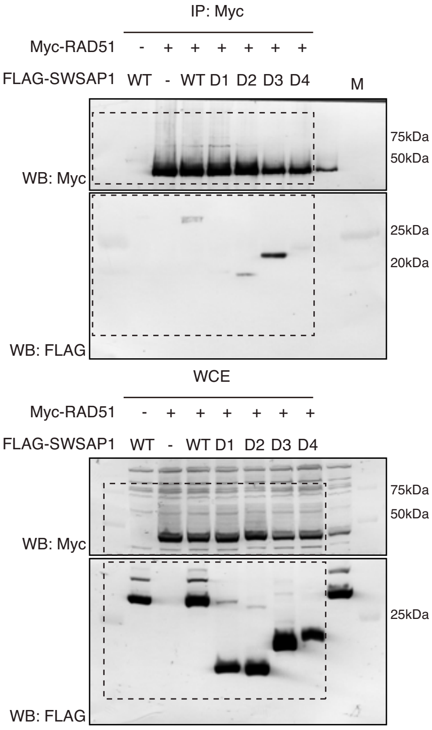

Related to Fig.1e

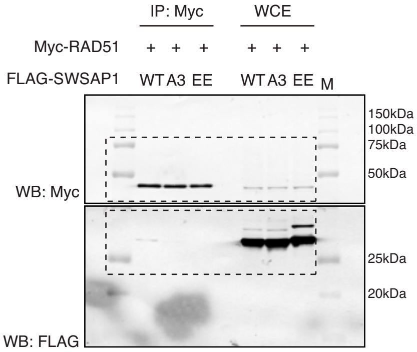

Related to Fig.2a

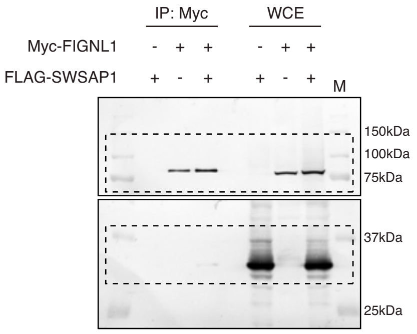

Related to Fig.2b

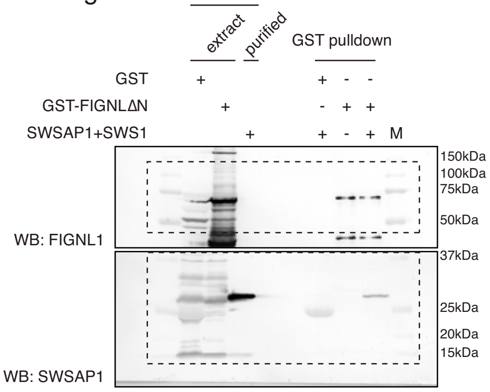

Related to Fig.2c

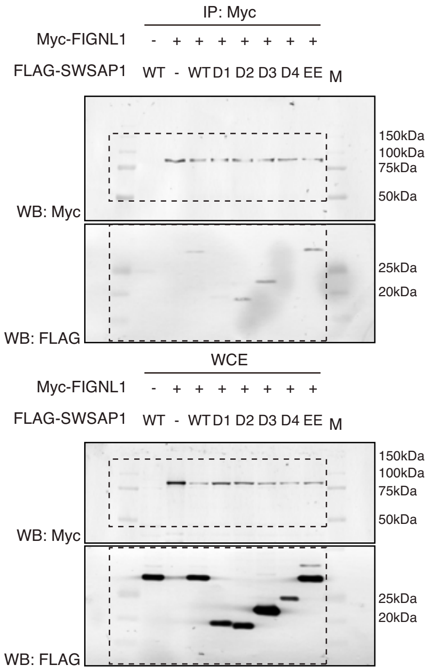

Related to Fig.2e

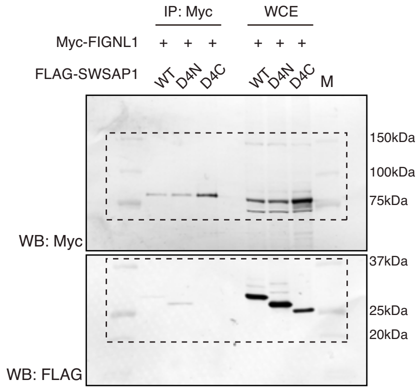

Related to Fig.2f

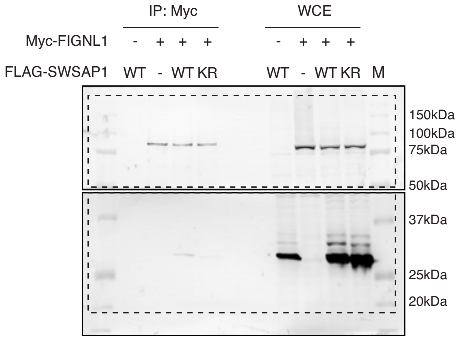

Supplementary Fig. 6a Uncropped scans for western blots and CBB staining

Supplementary Figure 6b

Related to Supplementary Fig.1e

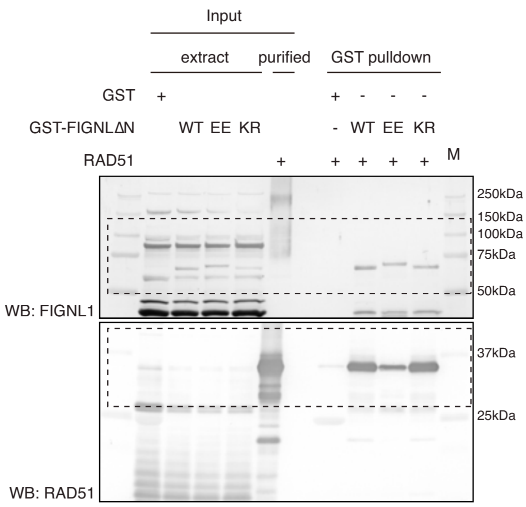

Related to Supplementary Fig.1f

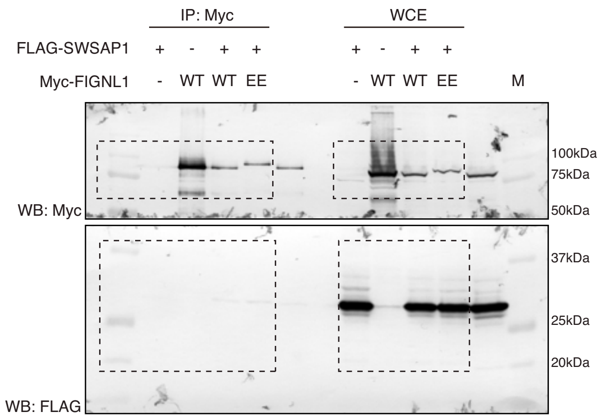

Related to Supplementary Fig.2b

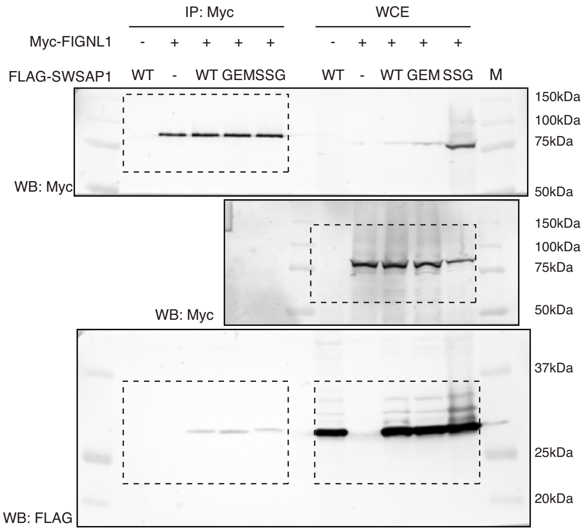

Related to Supplementary Fig.2c

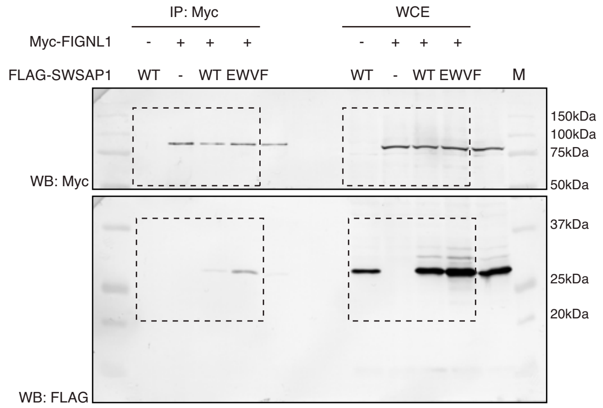

Related to Supplementary Fig.2d

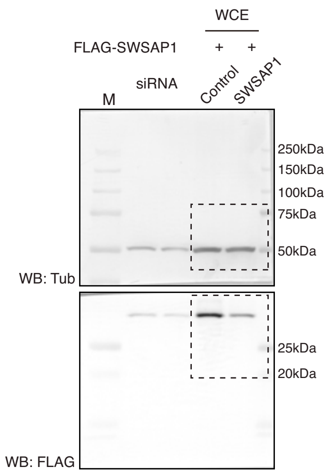

Related to Supplementary Fig.2e

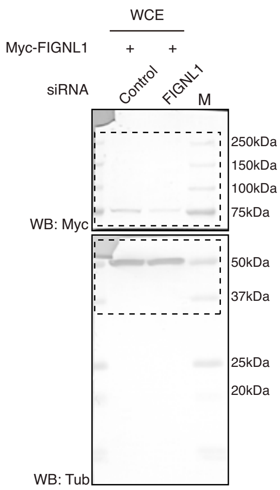

Related to Supplementary Fig.2f

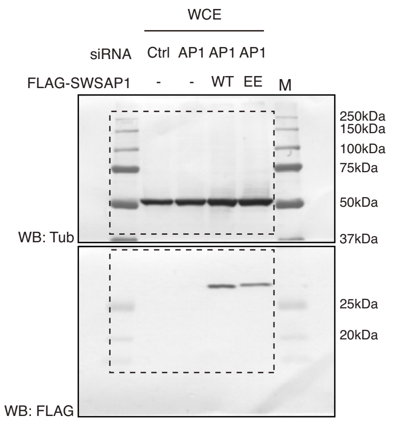

Related to Supplementary Fig.2g

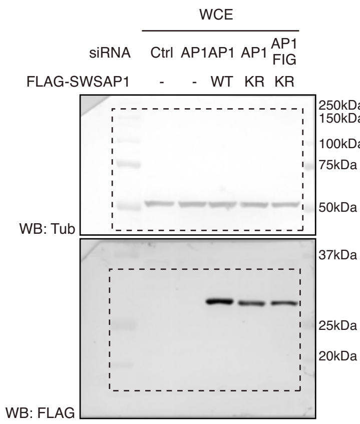

Supplementary Fig. 6b Uncropped scans for western blots and CBB staining

Supplementary Figure 6c

Related to Fig.4b

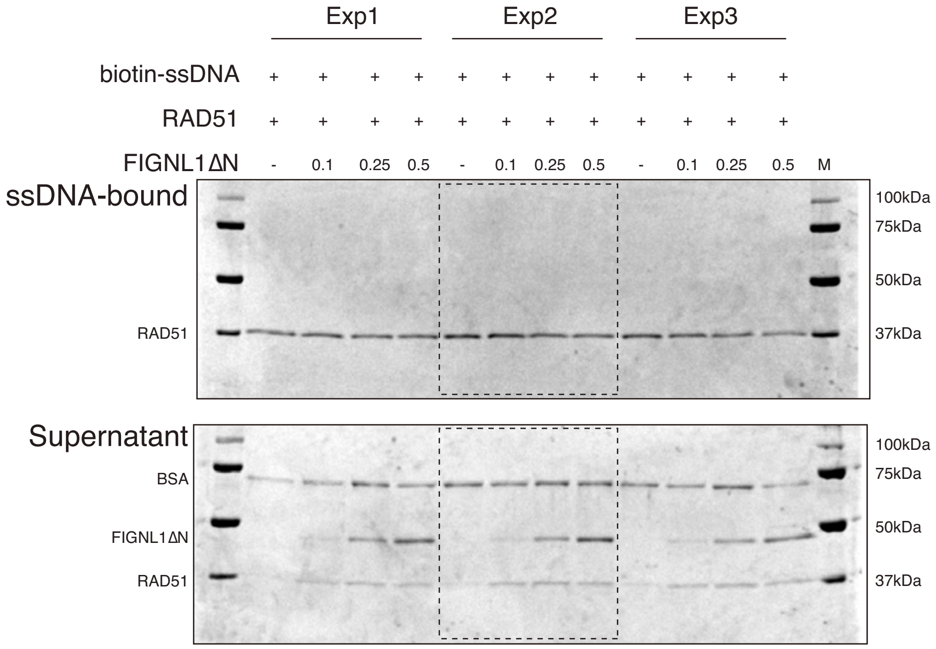

Related to Fig.4c

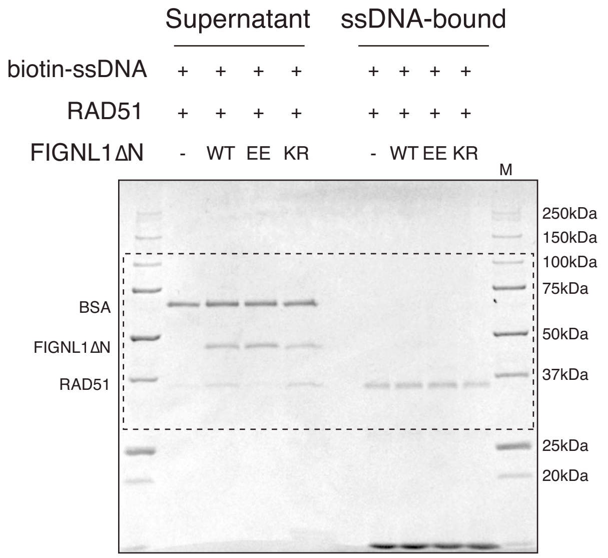

Related to Fig.4d

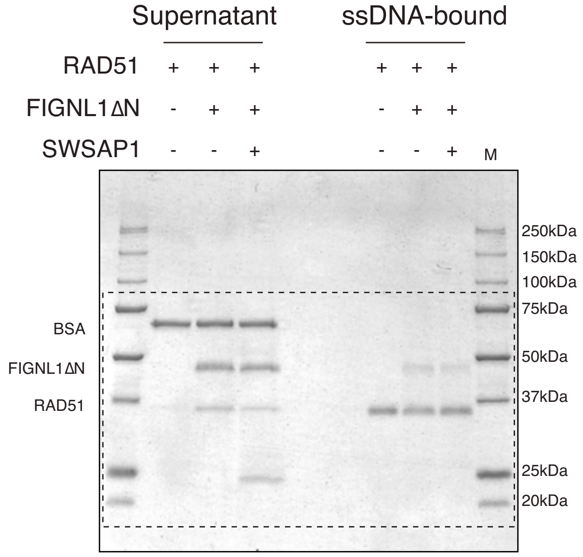

Related to Supplementary Fig.5a

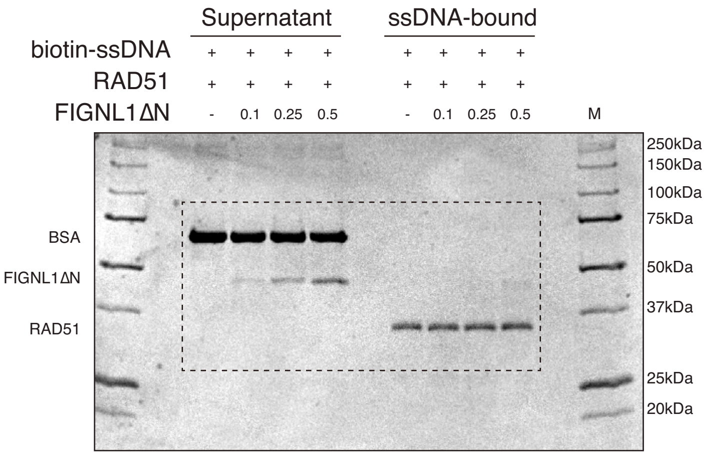

Related to Supplementary Fig.5b

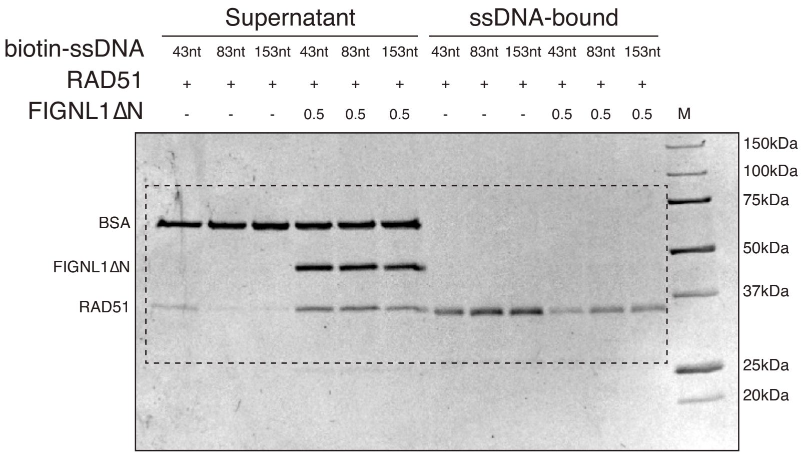

Supplementary Fig. 6c Uncropped scans for western blots and CBB staining
