# Supplementary material for: Human RAD51 paralogue SWSAP1 fosters RAD51 filament by regulating the anti-recombinase FIGNL1 AAA+ ATPase
Source: Nat Commun. 2019 Mar 29;10:1407. doi: 10.1038/s41467-019-09190-1 (PMC6440994; doi:10.1038/s41467-019-09190-1)
Supplement: Supplementary file 3 — Reporting Summary [file 41467_2019_9190_MOESM3_ESM.pdf]

nature research

Corresponding author(s): Akira Shinohara

Last updated by author(s): Dec 28, 2018

# Reporting Summary

Nature Research wishes to improve the reproducibility of the work that we publish. This form provides structure for consistency and transparency in reporting. For further information on Nature Research policies, see Authors & Referees and the Editorial Policy Checklist.

## Statistics

For all statistical analyses, confirm that the following items are present in the figure legend, table legend, main text, or Methods section.

| n/a | Confirmed | |
|---|---|---|
| ☐ | ☒ | The exact sample size (*n*) for each experimental group/condition, given as a discrete number and unit of measurement |
| ☐ | ☒ | A statement on whether measurements were taken from distinct samples or whether the same sample was measured repeatedly |
| ☐ | ☒ | The statistical test(s) used AND whether they are one- or two-sided<br>*Only common tests should be described solely by name; describe more complex techniques in the Methods section.* |
| ☒ | ☐ | A description of all covariates tested |
| ☒ | ☐ | A description of any assumptions or corrections, such as tests of normality and adjustment for multiple comparisons |
| ☐ | ☒ | A full description of the statistical parameters including central tendency (e.g. means) or other basic estimates (e.g. regression coefficient) AND variation (e.g. standard deviation) or associated estimates of uncertainty (e.g. confidence intervals) |
| ☐ | ☒ | For null hypothesis testing, the test statistic (e.g. *F*, *t*, *r*) with confidence intervals, effect sizes, degrees of freedom and *P* value noted<br>*Give P values as exact values whenever suitable.* |
| ☒ | ☐ | For Bayesian analysis, information on the choice of priors and Markov chain Monte Carlo settings |
| ☒ | ☐ | For hierarchical and complex designs, identification of the appropriate level for tests and full reporting of outcomes |
| ☒ | ☐ | Estimates of effect sizes (e.g. Cohen's *d*, Pearson's *r*), indicating how they were calculated |

*Our web collection on statistics for biologists contains articles on many of the points above.*

## Software and code

Policy information about availability of computer code

| | |
|---|---|
| Data collection | Image aquistion and deconvolution was performed using an image workstation (SoftWorks; Applied Precision) and iVision (Sillicon, USA) |
| Data analysis | Data were processed using iVision (Sillicon, USA), and Photoshop (Adobe, USA) software tools. GraphPad Prism 7 was used to assess statistical significance. |

For manuscripts utilizing custom algorithms or software that are central to the research but not yet described in published literature, software must be made available to editors/reviewers. We strongly encourage code deposition in a community repository (e.g. GitHub). See the Nature Research guidelines for submitting code & software for further information.

## Data

Policy information about availability of data

All manuscripts must include a data availability statement. This statement should provide the following information, where applicable:

- Accession codes, unique identifiers, or web links for publicly available datasets
- A list of figures that have associated raw data
- A description of any restrictions on data availability

*Provide your data availability statement here.*

# Field-specific reporting

Please select the one below that is the best fit for your research. If you are not sure, read the appropriate sections before making your selection.

☒ Life sciences ☐ Behavioural & social sciences ☐ Ecological, evolutionary & environmental sciences

# Life sciences study design

All studies must disclose on these points even when the disclosure is negative.

| | |
|---|---|
| Sample size | For biochemistry and cell biology, three independent experiments (biological) were used. For RAD51/DMC1 counting, more than 35 spreads were counted. For chromosome abnormality, more than spreads were counted. These (sample) numbers are accepted to the community. |
| Data exclusions | No data exclusion. |
| Replication | For biochemistry and cell biology, three independent experiments (biological) were used. |
| Randomization | For cell biology, cells were randomly divided to each treatment such as siRNA treatment. |
| Blinding | For cell biology, blinding was applied. On the other hand, biochemical experiments were carried out without blinding since exact combination of each reagent such as proteins and DNAs is important. In this case, multiple independent trials are one measure for reproducibility |

# Reporting for specific materials, systems and methods

We require information from authors about some types of materials, experimental systems and methods used in many studies. Here, indicate whether each material, system or method listed is relevant to your study. If you are not sure if a list item applies to your research, read the appropriate section before selecting a response.

## Materials & experimental systems

| n/a | Involved in the study | |
|---|---|---|
| ☐ | ☒ | Antibodies |
| ☐ | ☒ | Eukaryotic cell lines |
| ☒ | ☐ | Palaeontology |
| ☐ | ☒ | Animals and other organisms |
| ☒ | ☐ | Human research participants |
| ☒ | ☐ | Clinical data |

## Methods

| n/a | Involved in the study | |
|---|---|---|
| ☒ | ☐ | ChIP-seq |
| ☒ | ☐ | Flow cytometry |
| ☒ | ☐ | MRI-based neuroimaging |

## Antibodies

| | |
|---|---|
| Antibodies used | Anti-RAD51 (Santa Cruz, sc8364), anti-DMC1 (Santa Cruz sc22768), anti-SYCP3 (abcam, ab97672), anti-γH2AX (Millipore, 05-636) antibodies were used for immuno-staining for mouse spermatocyte spreads. Anti-RAD51 (Millipore, ABE257) and anti-γH2AX (Millipore, 05-636) antibodies were used for immunofluorescence staining of U2OS cells. For pulldown assay, anti-FIGNL1 (abcam, ab173685) and anit-SWSAP1 (Thermo, PA5-25460) were used. |
| Validation | Validation was initially based on online information from manufactures. Moreover the validity was independently evaluated by the authors. |

## Eukaryotic cell lines

Policy information about cell lines

| | |
|---|---|
| Cell line source(s) | U2OS |
| Authentication | None of cell used were not authorized/ |
| Mycoplasma contamination | The cell lines were not directly tested for contamination by Mycoplasma except direct microscope observation |
| Commonly misidentified lines (See ICLAC register) | N.A. |

## Animals and other organisms

Policy information about studies involving animals; ARRIVE guidelines recommended for reporting animal research

| | |
|---|---|
| Laboratory animals | Mus musculus, C57/BL6 |
| Wild animals | N.A. |

| | |
|---|---|
| Field-collected samples | N.A. |
| Ethics oversight | Animal care was conducted based on rules by Osaka university and performed in accordance with a protocol authorized by Institute for Protein Research, Osaka University, JAPAN. |

Note that full information on the approval of the study protocol must also be provided in the manuscript.
